# 454 sequencing put to the test using the complex genome of barley

**DOI:** 10.1186/1471-2164-7-275

**Published:** 2006-10-26

**Authors:** Thomas Wicker, Edith Schlagenhauf, Andreas Graner, Timothy J Close, Beat Keller, Nils Stein

**Affiliations:** 1 Institute of Plant Biology, University of Zürich, Zollikerstrasse 107, 8008 Zürich, Switzerland; 2 Leibniz Institute of Plant Genetics and Crop Plant Research, 06466 Gatersleben, Germany; 3 Department of Botany & Plant Sciences, University of California, Riverside, CA, 92521-0124, USA

## Abstract

**Background:**

During the past decade, Sanger sequencing has been used to completely sequence hundreds of microbial and a few higher eukaryote genomes. In recent years, a number of alternative technologies became available, among them adaptations of the pyrosequencing procedure (i.e. "454 sequencing"), promising a ~100-fold increase in throughput over Sanger technology – an advancement which is needed to make large and complex genomes more amenable to full genome sequencing at affordable costs. Although several studies have demonstrated its potential usefulness for sequencing small and compact microbial genomes, it was unclear how the new technology would perform in large and highly repetitive genomes such as those of wheat or barley.

**Results:**

To study its performance in complex genomes, we used 454 technology to sequence four barley Bacterial Artificial Chromosome (BAC) clones and compared the results to those from ABI-Sanger sequencing. All gene containing regions were covered efficiently and at high quality with 454 sequencing whereas repetitive sequences were more problematic with 454 sequencing than with ABI-Sanger sequencing. 454 sequencing provided a much more even coverage of the BAC clones than ABI-Sanger sequencing, resulting in almost complete assembly of all genic sequences even at only 9 to 10-fold coverage. To obtain highly advanced working draft sequences for the BACs, we developed a strategy to assemble large parts of the BAC sequences by combining comparative genomics, detailed repeat analysis and use of low-quality reads from 454 sequencing. Additionally, we describe an approach of including small numbers of ABI-Sanger sequences to produce hybrid assemblies to partly compensate the short read length of 454 sequences.

**Conclusion:**

Our data indicate that 454 pyrosequencing allows rapid and cost-effective sequencing of the gene-containing portions of large and complex genomes and that its combination with ABI-Sanger sequencing and targeted sequence analysis can result in large regions of high-quality finished genomic sequences.

## Background

Since the advent of genomic sequencing, technology has constantly been improved, leading to an approximately 3000-fold reduction in price per nucleotide sequenced [1]. The traditional method of sequencing is based on synthesis of a strand complementary to the template DNA with a reaction mix that contains dideoxy-nucleotides labelled with a fluorescent dye or a radioactive isotope [2]. Despite great progress in Sanger technology, alternatives were intensely sought to further decrease the sequencing costs and to approach the long-term goal of sequencing a genome for $1000 [1]. Recently, a new technology applying the principle of "pyrosequencing" [3,4] on a 'PicoTiterPlate™'-based reaction chamber was launched [5]. "Pyrosequencing" is a real-time "sequencing-by-synthesis" method promising similar accuracy like Sanger dideoxy sequencing [6]. The name of the company offering the new technology, 454 Life Sciences Corp., quickly became synonymous with the method which therefore meanwhile has been referred to as "454-sequencing" [7]. Throughout this manuscript, we will refer to sequences obtained through the use of Sanger dideoxy technology from either ABI 3700 and ABI 3730 sequencers as "ABI-Sanger sequences" and to those obtained by 454 pyrosequencing technology as"454 sequences".

For 454 sequencing, genomic DNA is mechanically sheared into fragments of a few hundred bp and linked to microbeads in a 1:1 ratio. The microbeads are captured in droplets (micelles) of an emulsion, which serve as PCR microreactors for template amplification. The microbeads are then distributed into a fibre-optic slide (PicoTiterPlate™) where the four DNA nucleotides are added in turns. Integration of a nucleotide into a DNA strand in one of the wells is translated into a light signal by the firefly enzyme luciferase (e.g. if adenins are added to the chain, light emission will occur only in those wells where an A is integrated).

The intensity of the signal is proportional to the number of nucleotides, if any, that are integrated in one step [5]. Utilising such a technological setup was shown to be highly effective for sequencing compact microbial genomes, which contain only very low amounts of repetitive DNA [5]. However, it is not known how 454 sequencing technology would perform on template derived from a large and highly repetitive genome such as that of barley.

Eukaryotic genome sizes vary enormously from 20 million base pairs (Mbp) in yeast to more than 127,000 Mbp in the lily *Fritillaria assyriaca *[8]. These differences are mostly attributable to repetitive DNA (e.g. transposons or tandem repeats). For example, the barley genome (5,500 Mbp) is almost twice the size of the human genome and contains more than 80% repetitive DNA [8]. To make large genomes accessible for sequencing, DNA is usually stored as bacterial artificial chromosomes (BACs) of 100–150 kb size.

ABI-Sanger and 454 sequencing protocols share few common steps but differ in many ways (Table 1) and a thorough comparison of the principles of the two sequencing procedures has been provided before [9]. In short: Common to both technologies is mechanical shearing of the target DNA (in our case BAC DNA) into fragments of 2–10 kb for ABI-Sanger and a few hundred bp for 454 sequencing. ABI-Sanger sequencing requires sub-cloning the sheared DNA fragments into *E. coli *cells (referred to as "shotgun library"). Individual clones have to be picked and grown in liquid media for propagation of plasmid DNA. Subsequent Plasmid DNA extraction provides the templates for the sequencing reaction. Modern ABI-Sanger sequencers produce reads of about 800–1,000 high-quality bases while 454 sequencing reads, thus far, only reach 100–200 bp. The time required for shotgun sequencing is directly proportional to the size of the DNA to be sequenced. For example, a BAC clone with a size of 100 kb requires about 600 shotgun clones which are sequenced from both ends to be sufficiently covered (7 runs on a 3730xl sequencer).

454 sequencing can use fragmented BAC DNA directly, thus, making the production of cloned shotgun libraries unnecessary. Independent of the technology used, raw sequences are assembled into sequence contigs which, in the finishing phase, are connected properly to the final sequence. In a highly repetitive genome such as the one from barley, the finishing phase is usually the most time consuming of the entire sequencing process.

The performance of 454 sequencing has been tested in compact microbial genomes and higher plant plastomes, which contain only very limited amounts of repetitive DNA [9-11]. Sequencing of preserved fragments of Mammoth genomic DNA is, the only application of 454 sequencing to a larger eukaryotic genome [12]. Problems with repetitive sequences were described to a limited degree [7] but so far no study focused specifically on the technological challenges of using 454 sequencing in large and highly repetitive (plant) genomes.

The present study addresses the question of whether 454 sequencing could be an efficient and cost-effective alternative to traditional ABI-Sanger sequencing in repetitive genomes. We re-sequenced two previously published barley BACs to compare results from the two technologies. Additionally, we sequenced two new BACs to have unbiased information on what specific problems might arise if one uses only 454 sequencing. We found 454 sequencing to be very suitable and efficient for covering the gene-containing portions at a high quality and we describe in detail the problems that occurred specific to sequencing DNA from repetitive genomes. Additionally, we developed analysis and annotation strategies to obtain very useable and partially finished working drafts for the BAC clones sequenced.

## Results and discussion

Using 454 sequencing technology, we sequenced four BAC clones from barley to different levels of sequence coverage, ranging from 16.8 to 66-fold (Table 2). BAC clones 773K14 and 519J4 were previously sequenced with the Sanger method on ABI 3700 and ABI 3730 capillary sequencers, respectively [13,14], and served as controls for coverage of gene space and repetitive sequences by 454 sequencing.

BAC 519J4 contains only two genes and is otherwise comprised almost exclusively of repetitive DNA. It contains several retrotransposons which are flanked by long terminal repeat (LTR) sequences of several hundred bp that add another level of repetitiveness. Thus, BAC 519J4 is one of the most repetitive barley BACs published so far. In contrast, BAC 773K14 contains four genes and, although it is comprised of ~70% known repetitive elements, the BAC itself contains only a few multicopy sequences. BACs 604D5 and 509D2 were sequenced for the first time to provide unbiased information on how well BACs can be sequenced and assembled using 454 sequencing. EST hybridisation experiments had indicated that these two BACs are gene-rich.

For all four BACs, we did two independent 454 sequencing runs, referred to as experiment 1 and 2 (Table 2) with experiment 1 resulting in about ten times more sequencing reads than experiment 2. Estimated sequence coverage ranges from 3.3-fold (BAC 509D2, experiment 2) to 66-fold (BAC 604D in experiment 1). For all experiments, 454 Life Sciences Corp. provided sequence assemblies. Assembled contigs as well as individual 454 reads were obtained in FASTA format. Additionally, SSF files (the equivalent of ABI chromatograms) were available.

**Figure 1:**
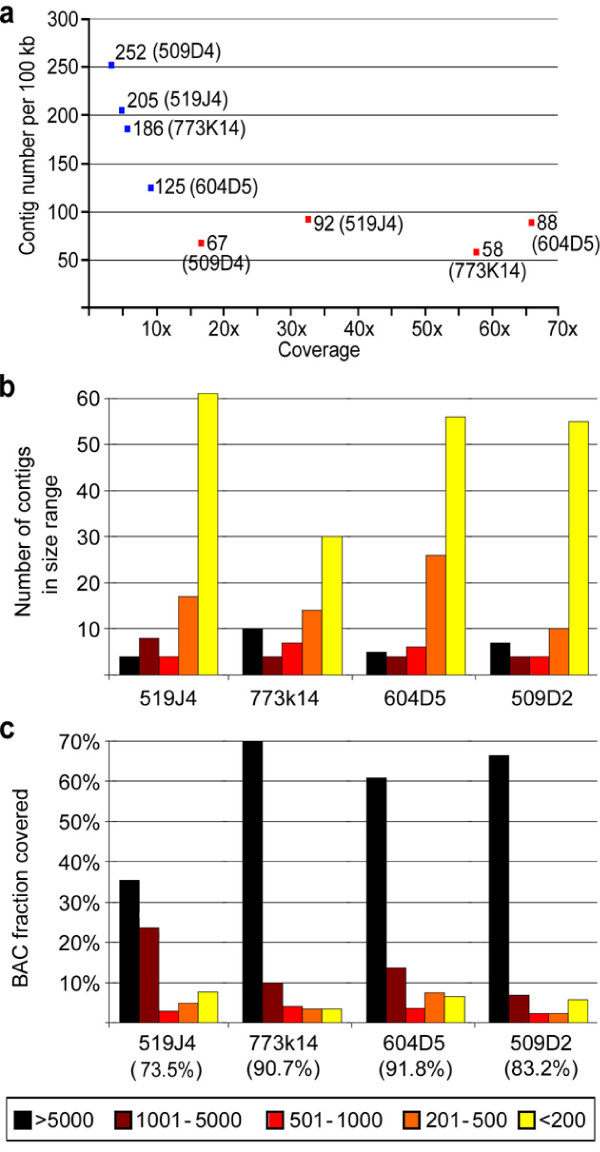
**Coverage of four BAC clones with sequence contigs assembled from sequence reads produced by 454 sequencing technology**. **a. **Relationship between coverage and number of sequence contigs from two independent sequencing experiments 1 (blue) and 2 (red) for all four BACs. Because the BACs have different sizes, the number of contigs is normalised. **b. **Numbers of sequence contigs in different size ranges from experiment 1. Assembly of 454 sequences resulted for all four BAC clones in a few large and many small sequence contigs. **c. **Percentage of the total size of the BACs covered by sequence contigs of different size ranges from experiment 1. The cumulative size of all contigs was in all four cases smaller than the actual size of the BAC clone (percentage in parentheses underneath the BAC name). This is due to pooling of repetitive sequences into consensus contigs. For BAC 604D5 and 509D2, the percentage was calculated based on size estimates from agarose gels.

Sequence reads from experiment 1 were assembled into 65 to 97 sequence contigs whereas experiment 2 resulted in 137 to 302 sequence contigs (Table 2). The assembly data from all four BACs show that the number of sequence contigs decreases rapidly with increasing coverage and appears to reach a plateau between 9.1 and 16.8-fold coverage (Figure 1a). These data indicate that coverage of BACs with 454 reads beyond 15-fold redundancy will not significantly decrease the final number of independent sequence contigs and singleton reads.

Because experiment 1 produced a higher number of sequences, all of our subsequent analysis was done on this dataset, unless stated otherwise. The sequence assemblies contain 64–96 gaps and are, thus, far from finished BAC sequences. In comparison, the initial assembly of 1,035 ABI-Sanger sequences from BAC 519J4 resulted in 12 sequence contigs (11 gaps). Indeed, due to the massively longer reads, it is virtually impossible to obtain such high numbers of gaps with ABI-Sanger reads. For example, if a BAC clone of 100 kb is covered with 100 ABI-Sanger reads of 900 bp each (0.9 × coverage), one would expect 99 gaps if the 100 reads were totally regularly distributed. But in reality, many of these reads will actually overlap and result in a massively reduced number of gaps.

The 454 sequence contigs range in size from 87 to 20,922 bp and 64 – 80% of the total sequences were assembled into contigs longer than 1000 bp. The majority of sequence contigs have sizes of less than 500 bp, but they contribute only 7 – 14% to the total BAC sequences. Additionally, the cumulative size of all sequence contigs did not reach the actual size of the BAC clones for any of the assemblies (Figure 1b and 1c) due to repetitive sequences being pooled into consensus contigs. As described below, these properties of the resulting sequence were not problematic in the context of cataloguing gene content in BACs but pose major problems if finished BAC sequences are needed.

### Gene space and other single-copy sequences are covered at a high quality by 454 sequencing

**Figure 2:**
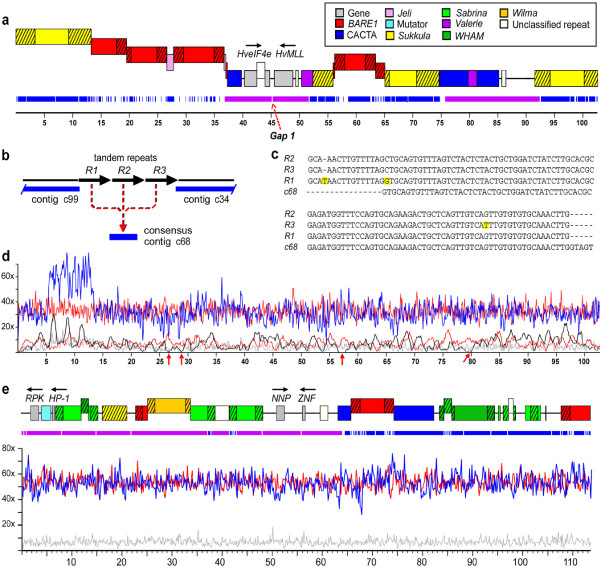
**Comparison of results from 454 sequencing with ABI-Sanger sequencing**. **a. **Map of the previously published BAC 519J4. Genes are depicted by grey boxes with transcriptional orientations indicated by arrows. Transposable elements are depicted as coloured boxes with LTRs indicated as shaded areas. Nested transposable elements are raised above the ones into which they have inserted. Regions covered by 454 sequence contigs are depicted as blue and purple bars underneath the map. Note that single copy sequences are covered well whereas multicopy sequences such as transposons or tandem repeats contain a large number of gaps. Sequence contigs used for comparison of ABI-Sanger and 454 sequencing results are depicted in purple. **b. **Detailed map of the region of *Gap1*. Three tandem repeats were pooled into the consensus contig c68. **c. **Multiple sequence alignment of the three repeat units shown in (b.) and the resulting consensus contig. Differences between repeat units are highlighted. **d. **Sequence coverage provided by 454 sequencing (blue) and ABI-Sanger sequencing (black). Red lines indicate simulated coverages with the same number of sequences assuming a purely random distribution. Red arrows indicate gaps in the ABI-Sanger coverage. Grey lines indicate coverage with 454 sequences from an independent sequencing experiment with fewer reads. The region of clearly higher coverage with 454 sequences suggests the presence of a duplicated sequence that could not be resolved with ABI-Sanger sequencing. **e. **Map of BAC 773K14 with aligned 454 sequence contigs and coverage with individual 454 sequences (colours as in **d**).

To study sequence quality, we compared the contigs assembled from 454 reads ("454 contigs") with the two previously published sequences of BACs 519J4 and 773K14. Three 454 contigs from BAC 519J4 and eleven from BAC 773K14 were compared with the published sequences (Figure 2). These 14 contigs have a cumulative size of 83,299 bp and cover mainly single copy regions of the two BACs. Over large stretches, the two technologies provided virtually identical results (56 differences, 99.93% identity) confirming the generally comparable level of accuracy provided by either pyrosequencing or Sanger dideoxy reads [6]. Forty differences occurred in stretches (homopolymers) of A or T. In all 40 cases, the stretches were one nucleotide longer in the 454 sequence, which is in contrast to previous findings that showed a tendency of homopolymers to be interpreted too short [5]. A survey of all A/T homopolymers in the analysed region showed that longer A/T stretches are more likely to cause problems (Table 3). An additional 7 differences were found one nucleotide away from a poly-A/T stretch whereas the other 9 had apparently random character. Surprisingly, there were no differences in the length of G/C homopolymers, although these are known to be problematic for ABI-Sanger sequencing. Assuming the same error rates for the two newly sequenced BACs 509D2 and 604D5, one can expect about 57 and 40 sequencing errors caused by A and T homopolymers, respectively, per 100 kb BAC.

Mapping of the 454 sequence contigs to the previously published BAC sequences also showed that gaps between sequence contigs are often only a few bp in size. In some cases, the gaps had size zero because two non-overlapping contigs mapped immediately adjacent to each other. Blast search of the gap-containing regions showed that many gaps were actually covered by multiple 454 sequences. For four gaps with sizes 0 or 1, we could show that they were caused by poly A/T stretches of 9–12 bp. All 454 sequences covering these gaps had low quality values in the A/T homopolymer, which is probably the reason why the motif was not accepted for the assembly. This indicates that some gaps may merely be a consequence of the stringency of the assembly method rather than truly missed sequences.

Apparently, stretches (homopolymers) of A and T pose the main problem in low copy regions. These findings are similar to those previously reported for 454 sequencing of plastid genomes [9]. If one excludes differences in A/T homopolymers and those found immediately next to such motifs, then the two technologies differ in only 9 positions which equals slightly more than 1 difference every 10,000 bp. We consider this to be an excellent match between the results of 454 sequencing and ABI-Sanger technologies. However, assuming that A/T homopolymers are the most abundant repeat sequence motifs in most genomes, efforts should be undertaken to improve the accuracy for these in 454 sequencing. It is perceivable that adjustments in the interpretation of signal intensity could significantly improve the sequence quality of homopolymers.

### Repetitive DNA is more problematic for 454 sequencing than for ABI-Sanger sequencing

Repeats such as LTRs or entire multicopy transposons were very poorly covered by the assembled sequence contigs of all four BACs because sequences from different copies of repeats were pooled and assembled into "consensus contigs" (Figure 2). In principle, 454 sequencing has the same problem as ABI-Sanger sequencing but due to the shorter read lengths, sequence pooling already occurs with motifs that are only little longer than 100 bp. If sequence pooling occurs only in highly repetitive DNA such as transposable elements, assembly of gene space can still easily be achieved at high quality. However, repetitive motifs in genic regions also can cause discontinuity in their assembly. For example, the short stretch between the two genes *HveIF4e *and *HvMLL *on BAC519J4 contains three tandem repeats of 144 and 145 bp, respectively (Figure 2b and 2c). With longer ABI-Sanger reads, this region is not problematic whereas in the assembly made from 454 sequences, the three repeat units were collapsed into one consensus contig, causing a gap in the otherwise completely assembled gene space (Figure 2a).

Any type of sequence at or below this size threshold of around 100 bp that occurs in multiple copies on a single BAC is problematic for 454 sequencing, independent of the copy number of the DNA elements in the whole genome context. A low-copy sequence that is duplicated locally might be an obstacle that would be hard to overcome whereas a transposable element that has 10,000 copies in the whole genome might not pose any problem if only a single copy would be present on the BAC clone of interest. Thus, sequence pooling might be a problem if genic regions containing repetitive motifs are targeted (e.g. gene family members, duplicated genes). Consequently, the determining factor for whether a sequence is covered well by 454 sequencing is its copy number on the sequenced BAC. Thus, even repetitive elements that are frequently found in or near genes (e.g. MITEs) do not cause problems in the assembly as long as they are present in only one copy on the BAC.

### Coverage with 454 sequences is more even than with ABI-Sanger sequences

To study coverage of BACs with 454 sequences and ABI-Sanger sequences, we used all individual raw sequences of BACs 519J4 and 773K14 in BLASTN searches against their published sequences to determine which part of the BAC clone was covered by each individual sequence. The total of that dataset allowed a visual representation of the overall coverage of the two BAC clones (Figure 2d and 2e). For comparison, sequence coverage was simulated assuming a purely random distribution of the same number of sequences of the same average sizes (Figure 2d and 2e). Over most of the BAC lengths, the coverage with 454 sequencing is very even, oscillating around an average value, and is virtually indistinguishable from the result of the simulation (Figure 2d and 2e). Except for a putative duplication (see below), there is no obvious difference in coverage of genes and transposable elements in either of these two BACs.

For BAC 519J4, the original ABI-Sanger raw sequences were also available and could be used for comparison (Figure 2d). Coverage with ABI-Sanger sequences shows large fluctuations and leaves four gaps which are not covered at all (Figure 2d). The simulation for ABI-Sanger sequencing shows a smaller variation and left a total of only 3 gaps during 50 repetitions of the simulation. The large fluctuations could be an effect of the cloning process which might discriminate against certain sequences. Since 454 sequencing does not require in vivo propagation of sub-fragments, replicative or recombinational incompatibilities are minimized. Interestingly, BAC 519J4 shows a region with clearly higher coverage by 454 sequencing; this suggests the presence of a duplication that was not resolved in the original ABI-Sanger sequencing effort (Figure 2d).

The coverage of BACs 519J4 and 773K14 with sequences from experiment 2 is very even, despite the fact that coverage was much lower for both BACs (Figure 2d and 2e). For all four BACs, we specifically tested how well the gene space was covered by sequence contigs from experiment 2. Here, we defined gene space as the coding region plus 1.5 kb upstream and 1 kb downstream. For the two BACs with the lowest coverage (509D2 and 519J4) of 3.3 and 4.9×, respectively, only 12% – 54% of the gene space was covered with 1 to 6 contigs. In contrast, on BAC 604D5 (9.1-fold coverage), more than 99% of the gene space was covered by very closely spaced sequence contigs which left gaps of only a few bp. The BAC 773 gene space was represented by 64%–93% at a coverage of 6.5×. At all coverage levels, all genes were at least partially covered and no genes were completely missed.

The availability of ABI-Sanger sequences for BAC 519J4 allowed experiments with hybrid assemblies, which showed that the inclusion of only 100 ABI-Sanger sequences closed more than half of the gaps in the 454 contig assemblies (Table 4). Comparison with the published sequence showed that 454 sequence contigs were joined correctly by ABI-Sanger sequences in most cases. Thus, a strategy which combines 454 sequencing with low-pass coverage of ABI-Sanger sequences may be helpful in scaffolding and gap closure when finished BAC sequences are required. In a previous study, the reverse approach was described when adding the data of one or two 454 sequencing runs to a 5.3-fold coverage by ABI-Sanger sequences was used as a strategy to increase quality and decrease costs in microbial genome sequencing projects [7].

### Useful BAC draft sequences can be assembled easily from 454 sequences

The two newly sequenced BACs 604D5 and 509D2 were found to contain 6 and 5 putative genes, respectively, and about 60% repetitive DNA (Table 5). All gene containing (i.e. single- or low-copy) regions were assembled in sequence contigs of >10 kb whereas most repeats were found in small contigs of only a few 100 bp. Despite the numerous gaps, the linear order of several contigs could be inferred by combining repeat analysis, comparative genomics and use of low-quality 454 sequences, as follows.

**Figure 3:**
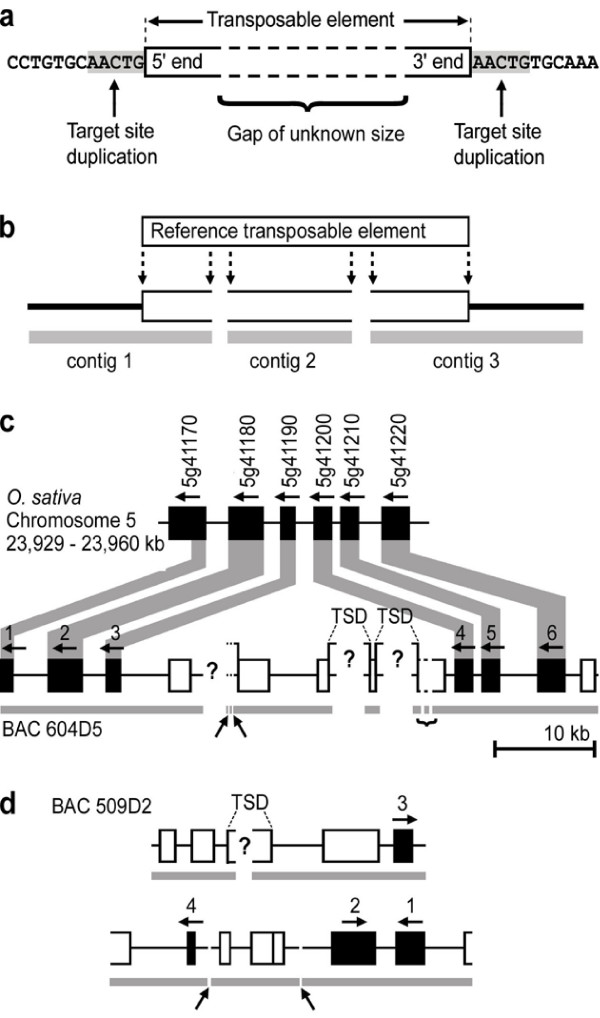
**Production of working drafts of BAC sequences from assemblies of 454 sequences**. The relative order of sequence contigs can be inferred through (**a.**) identification of target site duplications (TSD) of transposable element sequences located at the edges of contigs or (**b.**) sequence alignment with a known reference transposable element. The latter only works reliably for elements that occur only once on the BAC analysed. **c. **For BAC 604D5, information from the order of genes in the orthologous region of the rice genome was used as well as the structure and organisation of transposable elements. **d. **Five contigs from BAC 509D2 could be arranged in two supercontigs whose linear orientaion to each other is unknown. Regions covered by 454 sequencing contigs are indicated as grey bars underneath the maps in **c. **and **d.**. Genes are depicted as black and transposable elements as white boxes. Transcriptional orientations of genes are indicated by arrows. TSD used to infer contig order are indicated. Gaps that were closed through alignment to reference transposon sequences are indicated by a curly bracket. Gaps that could be closed with low-quality 454 sequences are indicated by upward arrows. Question marks indicate a gap of unknown size between. Numbers above genes correspond to gene descriptions in Table 5.

The ends of multicopy transposable elements were often found at the outer edges of large sequence contigs, which allowed inference of contig order by identifying matching target site duplications (Figure 3a). Transposable elements are usually flanked by 2–9 bp target site duplications (TSD) which are generated during their integration into the genome. If the 5' and 3' ends of a single known transposable element are found on different sequence contigs and both are flanked by the same TSD, one can assume that the two ends belong to the same element. Thus, in such instances the likely linear order of the sequence contigs can be inferred without precise knowledge of the size and sequence of the gap that separates them. For transposons that occurred only once on the BAC, the linear order of 454 sequence contigs was deduced through alignment to reference transposon sequences (Figure 3b). In BAC 604D2, six sequence contigs could be arranged through identification of target site duplications and alignment with reference transposon sequences (Figure 3c) whereas in BAC 509D2, a CACTA transposon was used to connect two large sequence contigs (Figure 3d).

Between grass species, the linear order of genes is often conserved, reflecting their descent from a common ancestor [15,16]. In the case of BAC 604D5, two large contigs containing three genes each showed perfect colinearity with the corresponding region of rice chromosome 5 and, thus, could be arranged in their likely linear order (Figure 3c). In BAC 509D2, only two genes are colinear in rice and both were already placed on the same sequence contig.

Additional clues as to the linear order of sequence contigs were obtained from 454 sequences which bridge some gaps but were not included in the assembly due to motifs with low sequence quality (see above). A combination of the three approaches allowed the linear arrangement of 48 kb of sequence contigs for BAC 604D2 whereas BAC 509D2 could be arranged into two supercontigs of 35.5 kb and 24 kb, respectively (Figure 3c and 3d).

## Conclusion

The dataset presented here, although relatively small, has convinced us that 454 sequencing could provide an efficient alternative to ABI-Sanger sequencing even if sequences of a complex or repetitive genome are targeted. An important finding of this study is that for all four BACs, 454 sequencing technology provided an excellent coverage of all gene containing fractions already in the initial sequence assemblies. The four BACs contain a total of 17 putative genes and at least the coding sequences of all genes were covered completely by 454 sequence contigs. For most genes, up- and downstream sequences were also present on the same sequence contig. Since genic sequences are usually the regions of the highest interest, the four BACs can be considered sufficiently covered.

As long as finished sequences are not imperative, 454 sequencing can provide advantages in cost and time over classical ABI-Sanger sequencing. In the present study 454 sequencing of 4 BACs covered by a single full 454 sequencing run (13000 USD) was approximately 2-fold less expensive than by ABI-Sanger sequencing the individual clones to 6-fold coverage (5000 USD each). This direct comparison, however, is very much dependent on local personnel costs and capacity of the chosen sequencing facility/provider. The most profound cost factor in the comparison of both approaches is time: 20 Mb of sequences are obtained in a single 454 sequencing run taking about 4 h [5]. Depending on the availability of the setup this would provide 20-fold coverage and thus full gene content information of 10 BACs (100 kb insert length) in a single 454 run. On the contrary, to achieve 2-fold coverage by ABI-Sanger sequencing (enough to sufficiently cover gene space) of the same number of BACs would take 10 times longer if running a single 96 capillary ABI3730xl device. Costs for ABI-Sanger sequencing have decreased ~1,000-fold over the last 15 years [1]. Although speculative, a similar development may be anticipated for pyrosequencing costs given a broader acceptance and use of high-throuput pyrosequencing, thus, inexpensive and rapid sequencing of large and complex genomes may soon enter a new era.

Due to the difficulties described in the assembly of repetitive sequences, a whole-genome shotgun approach by 454 sequencing does not seem practical for multi-gigabase plant genomes. Rather, a BAC-by-BAC approach, or perhaps small pools of BACs representing ~0.5 to 2.0 Mb contigs, may be the optimal formula for genomic sequencing in large and complex genomes. Our data show that at 9 to 10-fold coverage, the gene content of sequenced BACs will be completely revealed – even at lower coverage all genes contained on the clones will be at least partially hit. Thus, at a capacity of 20 Mb sequence obtained during a single 454 sequencing run, a 2.0 Mb contig represented as pool of individual BACs could be sequenced to 10-fold coverage with a high probability of detecting all of the genic sequences. For purposes such as the acceleration of map-based cloning and development of markers for marker assisted selection, this level of sequence resolution, if available genome-wide, would be a tremendous leap forward. If the genes of barley are indeed generally concentrated into gene-rich "islands" as suggested before [17-20], perhaps only about 1000 to 2000 contigs of an average size of 1 Mbp would need to be sequenced as BAC pools to collect most of the genic sequences of the barley genome. If so, then it should be possible within a 10 million dollar genome sequencing project, at today's costs, to apply 454 sequencing technology to a complete Triticeae genome. Considering current efforts to establish large BAC contigs that are anchored to genetic maps and cover nearly all of the barley genic regions within the next several years, it appears that barley is well positioned to serve as a proof of concept organism for such a venture.

Obtaining completely finished BAC sequences from a repetitive BAC clone using only 454 technology might by very problematic and time-consuming. It is perceivable that many gaps could be closed by designing primers at the ends of sequence contigs and using them for direct sequencing on the BAC clone. However, the several dozens of gaps in the initial 454 sequence assemblies would require an equal number of primers. Shotgun sequencing of BACs using ABI-Sanger sequencing has the advantage that information from forward and reverse reads of shotgun clones can help infer the linear order of sequence contigs. 454 sequencing so far, although under development [7], cannot provide such information. For highly repetitive genomes, the finishing phase is time- and labour intensive. Therefore, the choice of sequence strategy is crucial and our proposed combination of 454 with a small number of ABI-Sanger sequences seems promising.

In summary, we believe that our results describing a strategy combining detailed comparative genomics, refined repeat element analysis, the utilization of low-quality 454 sequences and taking advantage of low-pass ABI-Sanger sequences can lead to very useable working drafts of large and complex plant genomes in the near future.

## Methods

BAC clones were obtained from the Morex barley BAC library [21]. DNA was isolated with the QIAGEN large construct kit, adjusted to 200 ng/μl and provided to 454 Life Science Corp. for 454 PCR template preparation [5]. Sequence reads, contigs and quality scores for sequences and contig were obtained from 454 Life Science Corp.

For sequence analysis, programs from the EMBOSS package [22], CLUSTALW [23] and DOTTER [24] were used. Pairwise sequence alignments were produced with the program EMBOSS program WATER using a gap creation penalty of 30.0 and a gap extension penalty of 0.1. Repetitive elements were identified by BLAST [25] against the database for Triticeae repetitive elements (TREP [26,27]). Genes were identified by BLASTX and BLASTN against all CDS and proteins from rice (version 3) and Arabidopsis (version 5) genomes obtained from TIGR [28] and annotated by hand.

For hybrid assemblies, 454 contigs for BAC 519J4 (94 contigs, ranging in lengths from 91 to 16,582 bp) were converted to artificial reads assigning a Phred quality score of either 20 or 40 to each base using the CONSED package [29]. Base calling of the 1,035 ABI-Sanger reads for BAC 519J4 was done using PHRED (v. 020425.c, [30]). A series of ABI-Sanger data subsets representing different coverages were randomly generated using an original Perl script. Hybrid assemblies of the 454 and ABI-Sanger sequences were done with PHRAP (version 0.990319 [31]). Assembled contigs were mapped to reference BAC sequences using the MUMmer package (version 3.18, [32]).

Coverage of BACs 519J4 and 773K14 with 454 and ABI-Sanger sequence reads was determined by BLASTN of all individual reads against the published BAC sequence. For each read, positions of the strongest BLASTN hit on the BAC were used for graphical representation of sequence coverage. Only BLASTN hits >80 bp and >96% sequence identity were used. For the processing of large numbers of BLAST outputs, Perl programs were written. Coverage with 454 and ABI-Sanger sequences was simulated by choosing random positions in an interval corresponding to the size of the BAC. For the simulation it was assumed that all raw sequences have the size of the average of all raw sequence. Visual representation was done with the Perl Tk module [33]. The source codes for all original Perl programs written for this study are available upon request.

All 454 contigs containing genes were completely annotated and submitted to GenBank under the accession numbers DQ995508 – DQ995513. All smaller contigs were not submitted due to their small size and highly fragmented nature. All sequence data that were not deposited in GenBank are available upon request.

## Authors' contributions

TW carried out most of the Bioinformatics analysis. ES generated various sequence assemblies. AG contributed to the design of the experiments and the writing of the manuscript. TJC identified the gene-dense BAC clones and participated in the design and coordination of the work. BK contributed to the design of the bioinformatic analysis and the writing of the manuscript. NS was the initiator and PI of the project. All authors took part of drafting, reviewing and approval of the final manuscript.

## Figures and Tables

**Table 1:** Comparison of ABI-Sanger and 454 sequencing procedures

	ABI-Sanger	454	time required^a^
Isolation of BAC DNA	x	x	1 day
Mechanical shearing	x	x	2 h
Cloning	x		4 h
Clone picking	x		2 h
Plasmid DNA extraction	x		20 h
Reactions on thermocycler	x		36 h
Clean up reaction products	x		2 h
ABI 3730xl sequencer run	x		24 h
454 sequencing library		x	4 h^b^
Amplification in PCR microreactors		x	6 h^b^
GS 20 sequencing run		x	4 h^b,c^
Assembly of raw sequences	x	x	days to weeks

^a^The procedures and estimated time requirements describe the process for sequencing a BAC clone with a size of 100 kb. For ABI-Sanger, numbers are calculated to reach an approximately 10-fold coverage.

^b^according to [5]

^c^One 454 GS20 run produces ~20 Mb, approximately 10 times more than required for a sufficient coverage (20 ×) of a 100 kb BAC clone.

**Table 2:** Sequence coverage of four BAC clones from two independent sequencing experiments using 454 sequencing technology

BAC	size (bp)	total reads^a^	avg. size (bp)^b^	contigs	contig size (bp)	coverage
773K14	113.510^c^	59.126^e^	101	65	109.444	52.7 ×
		6.108^f^	104	210	106.476	5.6 ×
519J4	102.554^c^	32.564^e^	102	94	75.526	32.7 ×
		4.917^f^	102	209	72.634	4.9 ×
604D5	110.000^d^	70.510^e^	103	97	101.195	66 ×
		9.683^f^	103	137	102.468	9.1 ×
509D2	120.000^d^	19.208^e^	105	80	100.062	16.8 ×
		3.801^f^	104	302	66.647	3.3 ×

^a^Sequences containing BAC vector or *E. coli *were removed.

^b^Average read length of 454 sequences.

^c^Previously published, exact size is known.

^d^Estimated by gel electrophoresis.

**Table 3:** Differences between ABI-Sanger sequences and sequence contigs assembled from 454 sequences in poly A or T homopolymers in 83,299 bp of compared sequence

Motif^a^	total occurrences^b^	differences	error rate
A5	242	8	3.3%
A6	90	10	11%
A7	33	11	33%
A8	16	8	50%
A9	7	3	43%
A10	2	0	0
A11	1	0	0
A13	1	0	0

^a^Also includes the complementary poly-T motifs.

^b^Number of motifs in the compared 89 kb region.

**Table 4:** Results from hybrid assemblies of BAC 519J4.

ABI-Sanger reads	total contigs^a^
50^b^	47, 48, 44, 47, 46
100^b^	43, 40, 33, 35, 40
100^c^	59, 47, 48, 52, 55
200^b^	40, 31, 35, 36, 40

The 94 sequence contigs provided by 454 Life Sciences Corp (454 sequence contigs). were combined with different numbers of ABI-Sanger reads randomly selected from a set of 1,035 reads. Each assembly was repeated 5 times with different randomly selected sets. Note that the assignment of Phred scale quality values of 40 to the bases in the 454 sequence contigs decreased the number of false collapses considerably while only slightly increasing the overall number of contigs.

^a^Number of contigs resulting from 5 repetitions of the assembly with 5 different randomly selected sets of ABI-Sanger sequences.

^b^Bases in 454 sequence contigs were artificially assigned Phred scale quality values of 20.

^c^Bases in 454 sequence contigs were artificially assigned Phred scale quality values of 40.

**Table 5:** Genes identified on the two newly sequenced BAC clones 604D5 and 509D2.

Index^a^	BAC	Rice homolog^b^	Description
1	509D2	Os01g70940	Potassium uptake protein
2	509D2	Os01g70950	Hypothetical protein
3	509D2	Os08g07830	Hypothetical protein
4	509D2	Os05g08460	putative F-box domain
5	509D2	Os05g01370	Polygalacturonase-inhibiting protein
1	604D5	Os05g41170	SET domain protein 105
2	604D5	Os05g41180	Proteasome subunit alpha
3	604D5	Os05g41190	Expressed protein
4	604D5	Os05g41200	Calmodulin
5	604D5	Os05g41210	Calmodulin
6	604D5	Os05g41220	Similar to GAL83 protein

^a^Numbers correspond to gene numbers in Figure 2.

^b^Identified by BLASTN.
